# miR-1323 suppresses bone mesenchymal stromal cell osteogenesis and fracture healing via inhibiting BMP4/SMAD4 signaling

**DOI:** 10.1186/s13018-020-01685-8

**Published:** 2020-06-29

**Authors:** Hui Xie, Ming Liu, Yaofeng Jin, Haiqing Lin, Yushan Zhang, Song Zheng

**Affiliations:** Department of Orthopedics, The Second Affiliated Hospital of Jiaxing University, No. 1518 Huanchengbei Road, Jiaxing, Zhejiang, 314299 China

**Keywords:** Fracture, Atrophic non-union, BMP4, SMAD4, miR-1323

## Abstract

**Background:**

Atrophic non-union fractures show no radiological evidence of callus formation within 3 months of fracture. microRNA dysregulation may underlie the dysfunctional osteogenesis in atrophic non-union fractures. Here, we aimed to analyze miR-1323 expression in human atrophic non-union fractures and examine miR-1323’s underlying mechanism of action in human mesenchymal stromal cells.

**Methods:**

Human atrophic non-union and standard healing fracture specimens were examined using H&E and Alcian Blue staining, immunohistochemistry, qRT-PCR, immunoblotting, and ALP activity assays. The effects of miR-1323 mimics or inhibition on BMP4, SMAD4, osteogenesis-related proteins, ALP activity, and bone mineralization were analyzed in human mesenchymal stromal cells. Luciferase reporter assays were utilized to assay miR-1323’s binding to the 3'UTRs of BMP4 and SMAD4. The effects of miR-1323, BMP4, and SMAD4 were analyzed by siRNA and overexpression vectors. A rat femur fracture model was established to analyze the in vivo effects of antagomiR-1323 treatment.

**Results:**

miR-1323 was upregulated in human atrophic non-union fractures. Atrophic non-union was associated with downregulation of BMP4 and SMAD4 as well as the osteogenic markers ALP, collagen I, and RUNX2. In vitro, miR-1323 suppressed BMP4 and SMAD4 expression by binding to the 3'UTRs of BMP4 and SMAD4. Moreover, miR-1323’s inhibition of BMP4 and SMAD4 inhibited mesenchymal stromal cell osteogenic differentiation via modulating the nuclear translocation of the transcriptional co-activator TAZ. In vivo, antagomiR-1323 therapy facilitated the healing of fractures in a rat model of femoral fracture.

**Conclusions:**

This evidence supports the miR-1323/BMP4 and miR-1323/SMAD4 axes as novel therapeutic targets for atrophic non-union fractures.

## Introduction

Atrophic non-union is a fracture healing failure showing no radiological evidence of callus formation within 3 months of fracture [1]. Various parameters contribute to the development of atrophic non-union, such as advanced age, smoking, diabetes, impairment in vascular supply, and excessive motion/instability at the fracture site [2]. Fracture non-union has also been associated with a decrease in bone mesenchymal stromal cell counts and a downregulation in their proliferative potential [3]. This is consistent with mesenchymal stromal cells’ potential for differentiation into osteoblasts [4] and their promotion of fracture healing [5]. The sequential transcriptional activation of a number of genes and extracellular bone matrix mineralization have been implicated in the osteogenic differentiation of mesenchymal stromal cells [6, 7]. Even though the significance of mesenchymal stromal cells in osteogenic differentiation has been established, their role in atrophic non-union is still unclear.

Bone morphogenic proteins (BMPs) have been implicated in osteogenesis; for instance, BMP2 and BMP4 regulate the differentiation of osteoblasts and bone formation [8]. Furthermore, the ability of a number of BMPs (e.g., BMP2, BMP4, BMP6, and BMP7) to promote osteoblastic differentiation in a number of mesenchymal precursor cell types has been demonstrated [9]. BMPs can be secreted from osteoblasts and can exert their effects through binding with corresponding cell receptors or with extracellular matrix proteins; extracellular BMPs can even exert paracrine functions [10]. Notably, the SMAD family of transcriptional factors are the chief downstream intracellular mediators of BMP signaling; various SMAD molecules have also been linked to osteoblastic differentiation in mesenchymal stem cells [11, 12]. This evidence suggests that dysregulation of BMPs and SMADs in mesenchymal stromal cells may be critical to dysfunctional osteogenesis in atrophic non-union fractures.

MicroRNAs (miRNAs) are short non-coding RNAs that inhibit gene expression post-transcriptionally by binding to the 3' untranslated regions (3'UTRs) of target gene mRNAs. Furthermore, miRNAs can regulate numerous processes, including cell migration, differentiation, proliferation, and death, in osteoblasts and mesenchymal precursor cells [13]. For example, miR-204/211 regulates RUNX2 in mesenchymal stromal cells, thus inhibiting osteoblastic differentiation [14]. Moreover, miR-26a downregulates SMAD1 levels in human adipose tissue-derived stem cells, subsequently inhibiting osteogenic differentiation [15]. Recently published microarray experiments have identified several differentially upregulated miRNAs in atrophic non-union fractures, including miR-1323, miR-27b-3p, miR-381-3p, miR-520d-5p, and miR-4694-3p [16]. This evidence suggests that dysregulation of these candidate miRNAs may underlie the dysfunctional osteogenesis in atrophic non-union fractures.

In this study, we selected the foregoing five candidate miRNAs for further analysis. Notably, our initial in silico analysis revealed that among these five miRNAs, only miR-1323 putatively targets the osteogenic differentiators BMP4 and SMAD4 [17, 18] and that miR-1323 also possessed the highest collective number of putative BMP family and SMAD family targets. Therefore, we chose to pursue further investigation on miR-1323. We analyzed the expression of miR-1323 in human atrophic non-union fractures and critically examined miR-1323’s underlying mechanism of action in primary human mesenchymal stromal cells. We found that miR-1323 downregulates osteogenic differentiation of human mesenchymal stromal cells via directly targeting BMP4 and SMAD4. We also demonstrated that miR-1323 antagomiR therapy is associated with improvements in fracture healing in a rat model of femoral fracture. This evidence supports the miR-1323/BMP4 and miR-1323/SMAD4 axes as novel therapeutic targets to improve healing of atrophic non-union fractures.

## Materials and methods

### Ethics statement

This research has been approved by the Institutional Review Board (IRB) of The Second Affiliated Hospital of Jiaxing University (Hangzhou, China). This work conformed to the tenets of the Declaration of Helsinki. Written informed consent was obtained from all tissue donors prior to participation. Animal experiments were conducted in accordance with the National Institutes of Health (NIH) Guide for the Care and Use of Laboratory Animals.

### Human fracture specimen collection

Our human studies involved atrophic non-union fracture specimens (*n* = 5) and standard healing fracture specimens (*n* = 5) collected during open reduction/internal fixation (ORIF). These specimens were derived from 10 unique, demographically matched adult Han Chinese male donors who had experienced a tibial fracture and had undergone ORIF. Atrophic non-union was post-operatively diagnosed and defined as a fracture healing failure demonstrating no radiological evidence of callus formation for three consecutive months following ORIF [1]. Exclusion criteria for tissue donors were as follows [19]: taking medication within 2 weeks preceding ORIF, septic non-union fracture, head injury, heavy alcohol use (defined as reporting consumption of > 4 drinks on any one day or > 14 drinks in any 1 week), liver disorders, arthritic/rheumatic disorders, malabsorption disorders, bone metabolic disorders, endocrine disorders (i.e., thyroid disease, osteoporosis, diabetes), chronic pulmonary disorders (i.e., asthma, emphysema/COPD), cardiovascular disease (i.e., angina pectoris, myocardial infarction, deep venous thrombosis), or systemic inflammation (plasma C-reactive protein (CRP) > 5 mg/l). Plasma CRP levels from fasted venous samples were measured by immunonephelometry using a Beckman special protein analyzer. All CRP measurements were above the lower detection limit of 0.15 mg/l.

### Human fracture specimen analysis

Samples were prepared for histopathological, immunohistochemistry (IHC), quantitative real-time PCR (qRT-PCR), Western blotting, and alkaline phosphatase (ALP) activity analyses from anonymized tibial fracture specimens as previously described with minor modifications [20]. qRT-PCR, Western blotting, and ALP activity analyses were performed as described in the relevant subsections below.

For histopathological analysis, tissue samples were fixed for 48 h in 4% paraformaldehyde, decalcified with 20% EDTA, and embedded in paraffin. Next, 4-μm sections were cut and stained with H&E or Alcian Blue. For IHC analysis, 4-μm sections of paraffin-embedded tissue were deparaffinized, rehydrated, and placed in a wash buffer bath according to the kit’s protocol (LSAB 2 System-HRP, Dako). Following trypsinization (0.15 mg/l) for 9 min in a phosphate buffer (pH 7.8), sections were incubated overnight (4 °C) with antibodies against BMP4 (1:100; ab39973, Abcam) or SMAD4 (1:100; ab40759, Abcam). BMP4 and SMAD4 staining were analyzed with a streptavidin-biotin immunoperoxidase technique (LSAB 2 System-HRP, Dako).

For light microscopy imaging (Leica DM2500, Wetzlar), a computer-assisted, true-color image analyzing system equipped with a digital camera (Leica DFC420, Leica) together with Qwin Plus (Leica Microsystem Imaging Solutions) were utilized.

### Mesenchymal stromal cells isolation and culture

Mesenchymal stromal cells were obtained from our institution’s cell bank and cultured as previously described [21]. Cultures were maintained at 37 °C in an incubator with 5% CO_2_. The standard medium was DMEM (Gibco) with antibiotics and 10% fetal bovine serum (FBS) (Invitrogen). Cells from the first three passages were used for subsequent experiments. The medium for osteogenic differentiation included α-MEM with 10 mM glycerophosphate, 50 mg/ml l-ascorbic acid, antibiotics, 10% FBS, and 100 nM dexamethasone (Sigma). Cells were incubated in this osteogenic differentiation medium for 7 days. Medium was replaced every 24 h. Analyses were conducted after this 7-day osteogenic differentiation period. For some experiments, the TAZ inhibitor verteporfin (2 μM, Sigma) or an equivalent volume of vehicle control (PBS) were added to the osteogenic differentiation medium over the 7-day osteogenic differentiation period [22, 23].

### Mesenchymal stromal cell miRNA transfection and lentiviral infection

For modulation of miR-1323 levels, mesenchymal stromal cells were transfected with miR-1323-3p inhibitor (80 nM), miR-1323-3p mimics (80 nM), or their respective negative controls (NC, 80 nM) using Lipofectamine RNAiMAX (Invitrogen). The oligonucleotide sequences are provided in Additional Table 1. Cells were incubated in osteogenic differentiation medium containing miR-1323 mimics/inhibitor for 7 days. Medium was replaced every 24 h. Analyses were conducted after this 7-day osteogenic differentiation period.

For stable overexpression of BMP4 or SMAD4, mesenchymal stromal cells were lentivirally infected with either BMP4, SMAD4, or NC sequence as previously described [24]. Briefly, the BMP4, SMAD4, or NC sequence clone (Dharmacon) was subcloned into the pNL-EGFP/CMV lentiviral (LV) overexpression plasmid to form LV-BMP4, LV-SMAD4, or LV-NC. The LV particles were then produced by transfection of HEK293 cells with the appropriate pNL-EGFP/CMV overexpression plasmid, the packaging plasmid pCMV_R8.9, and the envelope plasmid pMD2.G in a 3:2:1 ratio, respectively. After harvesting and concentrating by ultrafiltration, LV particles were aliquoted and stored at −80^0^C. After preparing cells under optimal conditions, cells were transferred into osteogenic differentiation medium and infected with LV particles at multiplicities of infection (MOIs, number of LV particles/number of cells) of 40, 20, 10, or 5.0 for 24 h to achieve moderate levels of BMP4 or SMAD4 overexpression. Cells were incubated in osteogenic differentiation medium for 7 days. Medium was replaced every 24 h. Analyses were conducted after this 7-day osteogenic differentiation period.

### Quantitative real-time PCR (qRT-PCR)

RNA extraction was carried out with TRizol (Invitrogen) and included DNase I treatment (Invitrogen). For cDNA synthesis, 1 μg total RNA was used with a reverse transcription kit (Takara). qRT-PCR was performed with SYBR Green according to established protocols on a Bio-Rad real-time PCR system. The primer sequences have been provided in Additional Table 2. Individual gene mRNA levels were normalized to GAPDH, while miRNA levels were normalized to RNU6B, using the 2^−ΔΔCt^ method.

### Western blotting

RIPA lysis buffer was used for protein extraction (Beyotime). Protein extracts were electrophoresed on 12% polyacrylamide gels and subsequently transferred onto PVDF membranes (Millipore). Next, 5% nonfat milk in TBST was used to block the membranes; subsequently, they were incubated with the following primary antibodies overnight at 4 °C: BMP4 (ab39973, Abcam), SMAD4 (ab40759, Abcam), ALP (sc-365765, Santa Cruz Biotechnology), collagen I (Col I, ab34710, Abcam), RUNX2 (sc-390351, Santa Cruz Biotechnology), TAZ (ab224239, Abcam), β-actin (cytoplasmic housekeeping, ab8227, Abcam), and Histone H3 (nuclear housekeeping, ab201456, Abcam). Then, membranes were incubated with species-appropriate, horseradish peroxidase (HRP)-conjugated secondary antibodies. An electrochemiluminescence kit was used for protein band visualization (Amersham).

### Alkaline phosphatase (ALP) activity assay

ALP activity in specimen homogenates or whole cell lysates was assayed with p-nitrophenyl phosphate (PNPP, Sigma) as previously described [25]. The level of PNPP was measured at an optical density (OD) of 405 nm with a UVmax Colorimeter (Molecular Devices). ALP activity was reported as the OD value per mg protein.

### ALP and Alizarin Red staining

ALP staining was utilized to analyze mesenchymal stromal cell function and differentiation. Cells were fixed for 30 s in 60% acetone/40% citrate, washed for 45 s with water, and incubated in darkness for 30 min in fresh staining solution containing Naphtol AS-MX phosphate and Fast Blue RR Salt.

Alizarin Red staining was used for measuring mineralization content. Briefly, cells were fixed in 0.4% formaldehyde (Klinipath) for 15 min, washed once with PBS and twice with water, and stained for 10 min with Alizarin Red (Sigma-Aldrich). With this method, calcium crystals were visualized in the matrix.

### Luciferase assay

The BMP4 3′UTR possesses one predicted binding site for miR-1323, while the SMAD4 3′UTR possesses two sites (site 1 and site 2). Therefore, BMP4 3′UTR, SMAD4 3′UTR (site 1), or SMAD4 3′UTR (site 2) fragments were PCR amplified and cloned downstream of *Renilla luciferase* in the psiCHECK2 vector (Promega) to make the wild-type (WT) luciferase reporter constructs (WT-BMP4, WT-SMAD4 (site 1), or WT-SMAD4 (site 2), respectively). Alternatively, the 3′UTR seed regions were mutated (MUT) to construct MUT-BMP4, MUT-SMAD4 (site 1), and MUT-SMAD4 (site 2) luciferase reporter constructs, respectively, as indicated in the figures.

HEK293 cells (ATCC) were grown to 60–70% confluence in 24-well plates then co-transfected with miR-1323 inhibitor or mimics along with 100 ng luciferase reporter construct using Lipofectamine 3000. Luciferase activity was determined after 48 h with the Dual Luciferase Reporter Assay System (Promega). Normalization to firefly luciferase activity was carried out.

### Rat model of femoral fracture

Male Sprague-Dawley (SD) rats were purchased from the SLACC Experimental Animal Center (Changsha, China). All rats were 13–14 weeks of age and within the weight range of 400–450 g. A model of femoral fracture was performed as previously published with slight modifications [26]. A transverse osteotomy was carried out in the mid-diaphysical section of the femur on day 0. Immediately thereafter, fracture reposition and stabilization via stainless steel pin implantation were performed; the pin was cylindrical (2 cm in diameter and 2.5 cm in length). On days 4, 7, and 11, either the negative control (NC) antagomiR or the miR-1323 antagomiR (GenePharma) was injected around the fracture site (5 nmol/100 μl) based on a previously published antagomiR dosing regimen [16]. The oligonucleotide sequences are provided in Additional Table 1.

On days 7 and 14, X-ray images of the fractures were performed using a standard X-ray device (GE Healthcare). The images were scored using the following scale described by Long et al. [16]: (1 point) no hard callus visible; (2 points) minor intramembranous ossification; (3 points) hard callus, no fracture gap bridging, apparent fracture line; (4 points) hard callus, fracture gap bridging, visible fracture gap; (5 points) unclear boundary between existing cortical bone and newly formed callus; or (6 points) remodeling. On day 14, the experimental animals were sacrificed, and fracture calluses were dissected out for further analysis.

### Statistical analysis

Data are expressed as means ± standard errors of the mean (SEMs) unless otherwise specified. Statistical analyses were carried out with GraphPad Prism. Prior to comparing groups, the normality assumption was validated using Kolmogorov-Smirnov tests. For continuous variables, Student’s *t* tests were employed for comparisons between two groups, while one-way ANOVA with post-hoc Tukey tests were used for comparisons among multiple groups. For discrete radiographic scores, comparisons were performed with χ2 tests. Pearson correlation analysis was applied to determine correlations between miRNA expression and protein expression. Statistical significance was set at *p* < 0.05 for all analyses.

## Results

### miR-1323 upregulation coupled with downregulation of BMP4/SMAD4 signaling and osteogenesis markers in atrophic non-union fractures

**Fig. 1:**
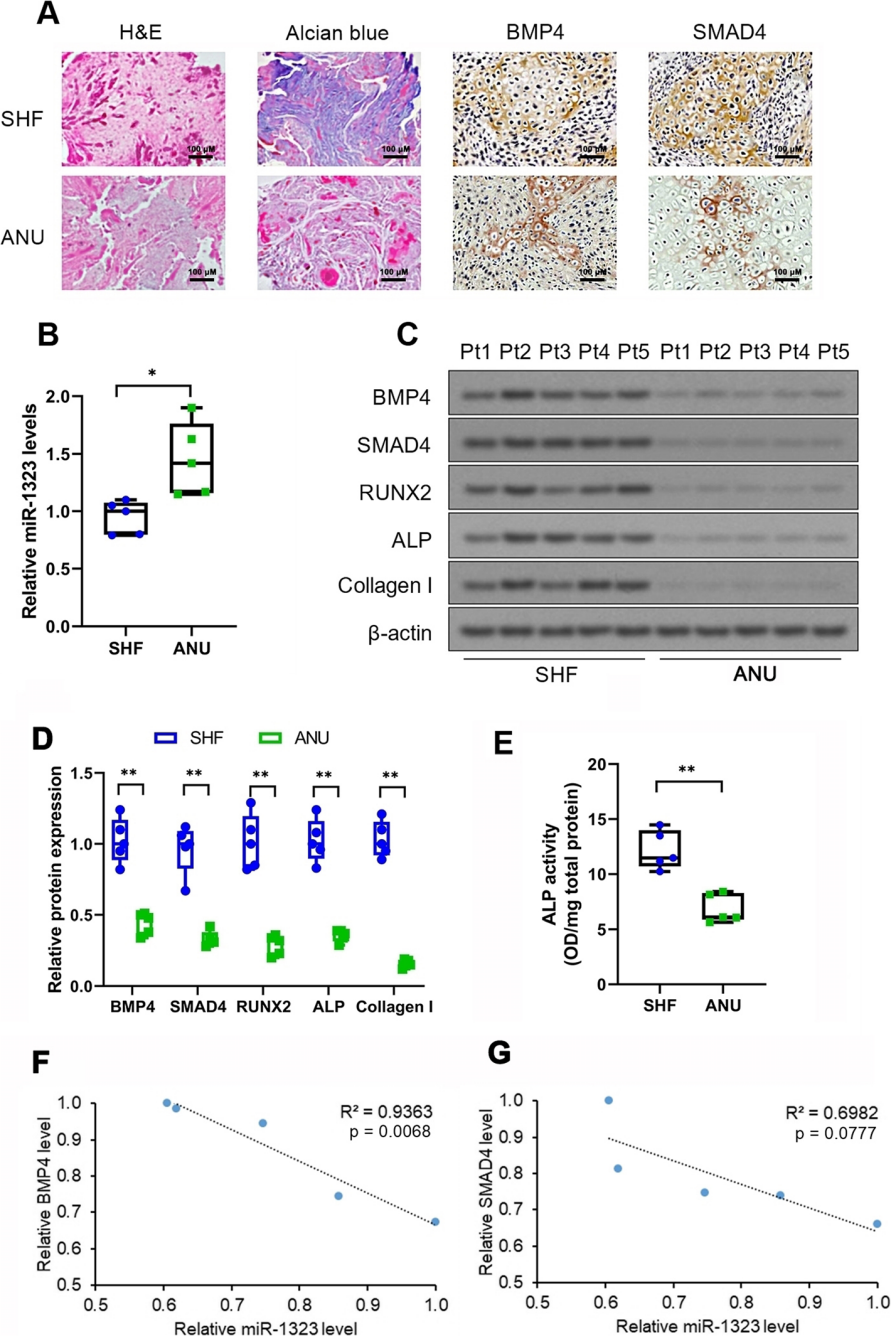
Downregulation of BMP4/SMAD4 signaling and osteogenesis-related markers in atrophic non-union fractures. **a** Histopathological analysis of atrophic nonunion (ANU) (*n* = 5) and standard healing fracture (SHF) specimens (*n* = 5) using H&E and Alcian Blue staining. IHC analysis of BMP4 and SMAD4 protein expression in ANU and SHF specimens. Scale bars = 100 μm. **b** qRT-PCR analysis of miR-1323 expression in ANU and SHF specimens. miR-1323 expression normalized to the SHF median value. **c** Representative immunoblots and **d** quantitation of BMP4, SMAD4, ALP, Col I, and RUNX2 protein expression in ANU and SHF specimens. Protein expression normalized to the SHF median values. **e** ALP activity in ANU and SHF specimens. ALP activity reported as the optical density (OD) 405 nm value per mg protein. **f**, **g** Pearson correlation analyses of (**f**) miR-1323 expression and BMP4 expression as well as (**g**) miR-1323 expression and SMAD4 expression in ANU specimens. *p < 0.05, **p < 0.01 [SHF vs. ANU]. **b**, **d**, **e** Data presented as medians ± interquartile ranges (boxes) and absolute ranges (whiskers)

The clinicodemographic characteristics of the human tissue donors are detailed in Additional Table 3. We first examined histopathological changes in atrophic non-union fracture specimens in relation to standard healing fracture specimens using H&E and Alcian Blue staining. The non-union specimens were characterized by fibrosis around the fracture fragments, neoangiogenesis, and a mild degree of new bone formation (Fig. 1a). As previously published microarray studies have suggested miR-27b-3p, miR-381-3p, miR-520d-5p, miR-1323, and miR-4694-3p upregulation in atrophic non-union specimens [16], we selected these miRNA species for further analysis. As human mesenchymal stromal cell osteogenic differentiation may be induced by BMP4, SMAD4, and SMAD4’s downstream mediators TAZ and RUNX2 [17, 18], we focused our initial TargetScan analysis on those miRNAs putatively targeting both BMP4 and SMAD4. We found that miR-1323 was the only species matching this criteria; moreover, miR-1323 had the highest collective number of putative BMP family and SMAD family targets (*n* = 15) as compared to miR-520d-5p (*n* = 14), miR-4694-3p (*n* = 14), miR-27b-3p (*n* = 8), or miR-381-3p (*n* = 3). Therefore, we chose to pursue further investigation on miR-1323.

qRT-PCR analysis validated miR-1323 upregulation in atrophic non-union fracture specimens (*p* = 0.02; Fig. 1b). IHC analysis of fracture specimens revealed lower BMP4 and SMAD4 expression in atrophic non-union tissues (Fig. 1a). Western blotting also showed that BMP4 and SMAD4 protein expression levels were downregulated in non-union fractures (*p* < 0.01; Fig. 1c, d). Furthermore, protein levels of ALP, Col I, and RUNX2—important markers of osteogenesis [27–29]—were reduced in non-union fractures (*p* < 0.01; Fig. 1c, d). ALP activity was downregulated in non-union fractures (*p* < 0.01; Fig. 1e). Pearson correlation analysis in the non-union fracture data a significant negative correlation between miR-1323 expression and BMP4 protein expression (*r* = −0.97, *p* < 0.01; Fig. 1f) as well as a negative correlational trend between miR-1323 expression and SMAD4 protein expression (*r* = −0.84, *p* = 0.08; Fig. 1g).

### The osteogenic differentiation of mesenchymal stromal cells is modulated by miR-1323

**Fig. 2:**
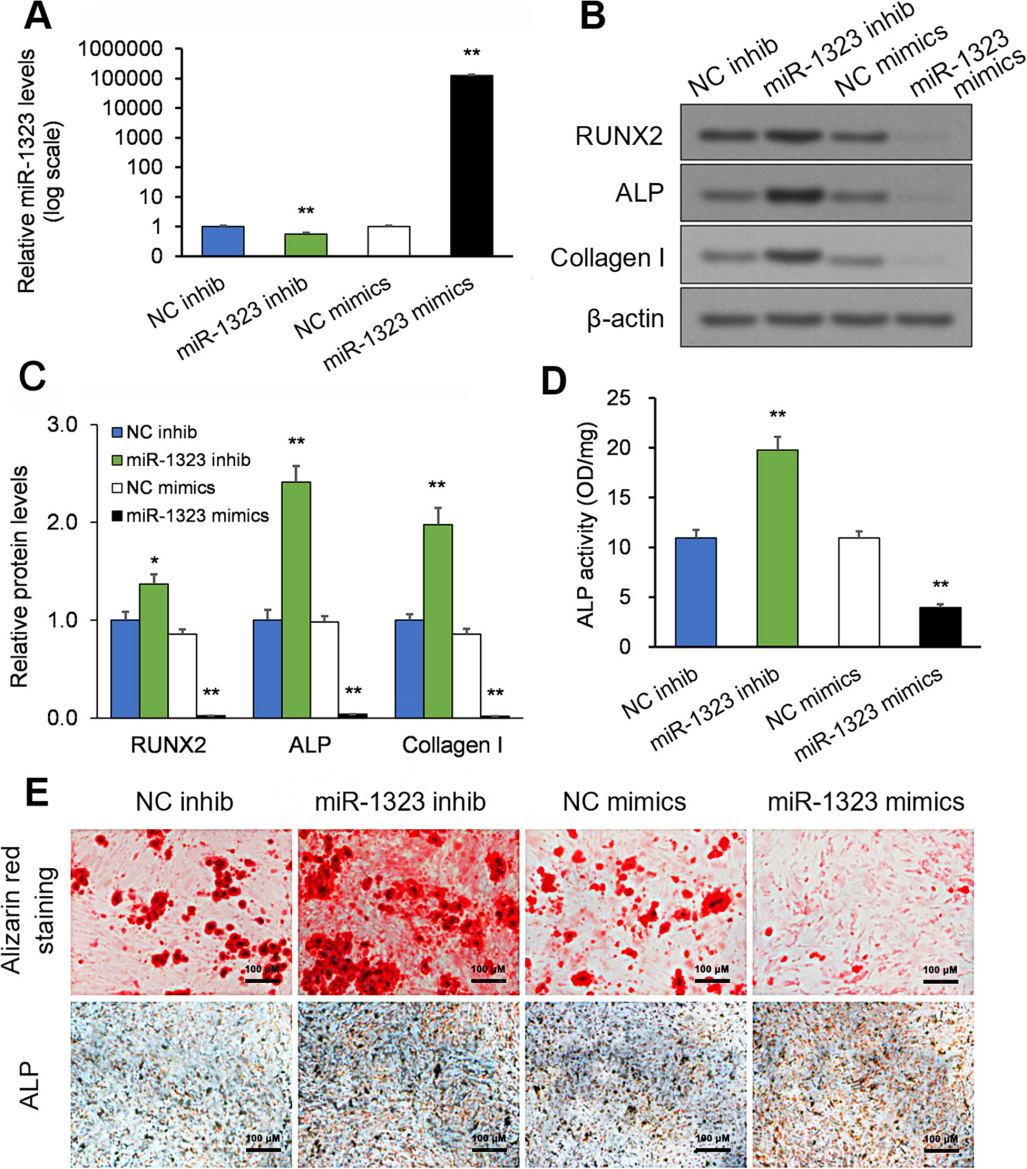
miR-1323 modulates mesenchymal stromal cell differentiation. Mesenchymal stromal cells were placed in osteogenic differentiation medium and transfected with miR-1323 mimics or inhibitors. **a** After 7 days of culture, qRT-PCR was used to confirm miR-1323 levels. **b**, **c** ALP, Col I, and RUNX2 protein expression was measured via Western blot. **d** The activity of ALP was measured. **e** Mineralization degree determined by staining with Alizarin Red and ALP. Scale bars = 100 μm. *p < 0.05, **p < 0.01 [miR-1323 inhib vs. NC inhib; miR-1323 mimics vs. NC mimics]. Data presented as means ± SEMs. All in vitro experiments: 3 biological replicates × 3 technical replicates

The effect of miR-1323 on osteogenesis-related proteins was analyzed in human mesenchymal stromal cells subjected to osteogenic differentiation. Cells in osteogenic differentiation medium were transfected with miR-1323 mimics or inhibitor. After 7 days of culture, miR-1323 downregulation by inhibitor and miR-1323 upregulation by mimics were validated by qRT-PCR (*p* < 0.01; Fig. 2a). miR-1323 inhibition upregulated ALP, Col I, and RUNX2 protein levels (RUNX2: *p* = 0.01; ALP, Collagen I: *p* < 0.01; Fig. 2b, c); the opposite effects were observed after miR-1323 mimics exposure (*p* < 0.01; Fig. 2b, c). ALP activity displayed a similar pattern in response to miR-1323 inhibition or miR-1323 mimics (*p* < 0.01; Fig. 2d). miR-1323 inhibition increased, whereas miR-1323 mimics, decreased bone mineralization (Fig. 2e). Thus, our evidence suggests that miR-1323 inhibition promotes mesenchymal stromal cell osteogenic differentiation.

### miR-1323 directly downregulates BMP4 and SMAD4 levels in mesenchymal stromal cells

**Fig. 3:**
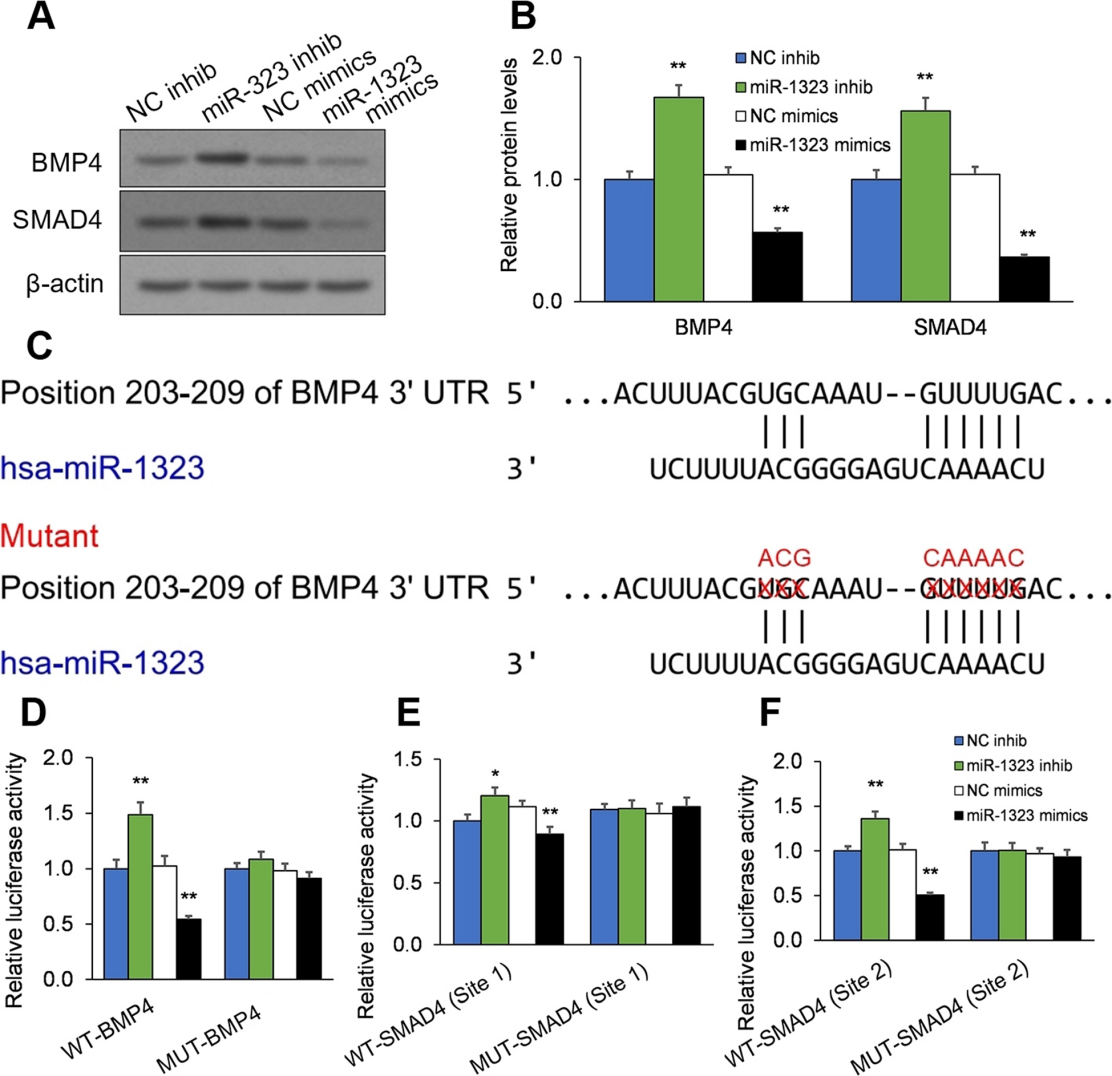
miR-1323 directly suppresses BMP4 and SMAD4 levels. **a**, **b** Human mesenchymal stromal cells were placed in osteogenic differentiation medium and transfected with miR-1323 mimic or inhibitor. After 7 days of culture, BMP4 and SMAD4 levels were measured by Western blot. **c** (Top) Wild-type miR-1323 binding site in the BMP4 3′UTR luciferase reporter construct (WT-BMP4 3′UTR) and (bottom) the engineered mutant miR-1323 binding site in the BMP4 3′UTR luciferase reporter construct (MUT-BMP4 3′UTR). **d** Luciferase reporter activity levels in HEK293 cells transfected with miR-1323 mimics or inhibitor along with the WT-BMP4 3′UTR or MUT-BMP4 3′UTR luciferase reporter construct. **e**, **f** Luciferase reporter activity levels in HEK293 cells transfected with miR-1323 mimics or inhibitor along with (**e**) the wild-type SMAD4 3′UTR luciferase reporter construct (WT-SMAD4 3′UTR) or mutant SMAD4 luciferase reporter construct (MUT-SMAD4 3′UTR) for site 1 or (F) WT-SMAD4 3′UTR or MUT-SMAD4 3′UTR for site 2. See Additional figure 1 for wild-type and engineered mutant site 1 and site 2 in WT-SMAD4 3′UTR and MUT-SMAD4 3′UTR. *p < 0.05, **p < 0.01 [miR-1323 inhib vs. NC inhib; miR-1323 mimics vs. NC mimics]. Data presented as means ± SEMs. All in vitro experiments: 3 biological replicates × 3 technical replicates

We next analyzed the effect of miR-1323 on BMP4/SMAD4 signaling in human mesenchymal stromal cells subjected to osteogenic differentiation. Human mesenchymal stromal cells were placed in osteogenic differentiation medium and transfected with miR-1323 mimics or inhibitor. After 7 days of culture, BMP4 and SMAD4 protein levels were determined by Western blot. BMP4 and SMAD4 expression were decreased and increased by miR-1323 overexpression and inhibition, respectively (*p* < 0.01; Fig. 3a, b).

Luciferase assays were utilized to elucidate whether miR-1323 directly binds to the 3′UTRs of BMP4 and SMAD4. Notably, the SMAD4 3′UTR possesses two putative miR-1323 binding sites (site 1 and site 2) (Additional Fig. 1). Therefore, wild-type (WT)-BMP4 3′UTR, WT-SMAD4 3′UTR (site 1), and WT-SMAD4 3′UTR (site 2) luciferase reporter constructs, as well as their mutant (MUT) counterparts, were designed (Fig. 3c, Additional Fig. 1). These vectors were co-transfected into HEK293 cells along with miR-1323 inhibitor or mimics, and the resulting luciferase activity levels were measured. miR-1323 inhibition promoted luciferase activity levels from the WT-BMP4 3′UTR construct (*p* < 0.01), WT-SMAD4 3′UTR (site 1) construct (*p* = 0.02), and WT-SMAD4 3′UTR (site 2) construct (*p* < 0.01) (Fig. 3d–f). In contrast, miR-1323 mimics suppressed luciferase activity levels from all three WT constructs (*p* < 0.01; Fig. 3d–f). Moreover, these luciferase activity changes were eliminated after mutations in the predicted miR-1323 binding sites (*p* > 0.05; Fig. 3d–f). Therefore, our evidence suggests that miR-1323 directly targets the 3′UTRs of BMP4 and SMAD4 and downregulates BMP4 and SMAD4 expression in mesenchymal stromal cells.

### miR-1323 modulates osteogenic differentiation of mesenchymal stromal cells via BMP4 and SMAD4

Next, the effects of miR-1323, BMP4, and SMAD4 expression were analyzed in human mesenchymal stromal cells subjected to osteogenic differentiation. Human mesenchymal stromal cells were placed in osteogenic differentiation medium and infected with LV-SMAD4 or LV-BMP4 and co-transfected with miR-1323 mimics. After 7 days of culture, the protein levels of BMP4, SMAD4, and osteogenesis factors were determined by Western blot. As expected, miR-1323 mimics downregulated BMP4, SMAD4, ALP, Col I, and RUNX2 protein levels (*p* < 0.01; Additional Fig. 2A, B). In contrast, BMP4 or SMAD4 overexpression upregulated protein levels of ALP (*p* < 0.01), Col I (LV-BMP4: *p* = 0.01, LV-SMAD4: *p* < 0.01), and RUNX2 (*p* < 0.01) (Additional Fig. 2A, B). Notably, the downregulating effects of miR-1323 mimics on ALP, Col I, and RUNX2 were rescued by BMP4 or SMAD4 overexpression (*p* < 0.01; Additional Fig. 2A, B). A similar pattern of findings were found with respect to ALP activity (*p* < 0.01; Additional Fig. 2C). Thus, miR-1323 modulates mesenchymal stromal cell osteogenic differentiation via regulating BMP4 and SMAD4 expression.

### miR-1323 regulates TAZ nuclear translocation via BMP4 and SMAD4, thus modulating osteogenic differentiation

**Fig. 4:**
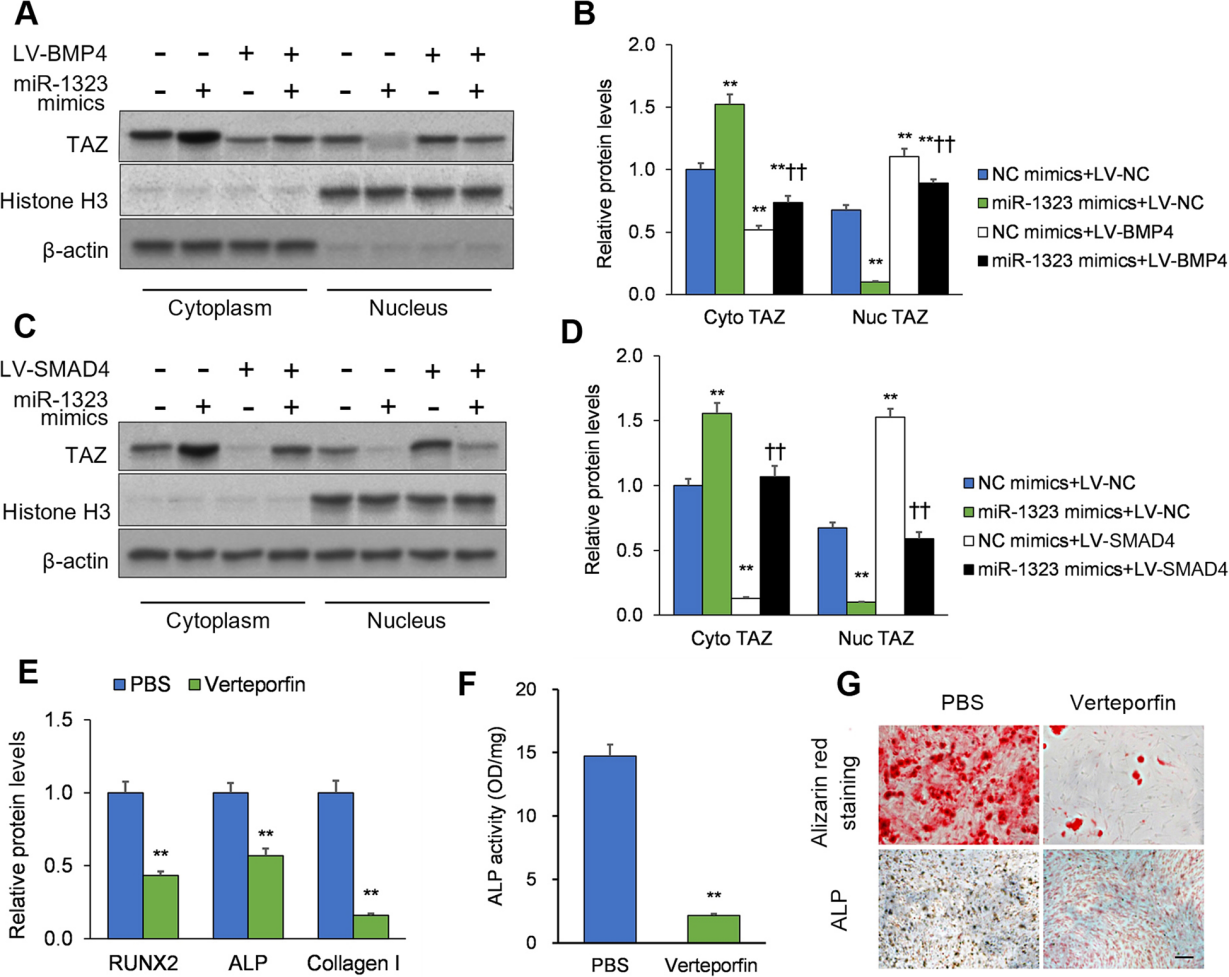
miR-1323/BMP4 and miR-1323/SMAD4 axes regulate osteogenic differentiation by modulating the nuclear translocation of TAZ. **a**-**d** Human mesenchymal stromal cells were placed in osteogenic differentiation medium and infected with LV-SMAD4 or LV-BMP4 and co-transfected with a miR-1323 mimics. After 7 days of culture, TAZ levels were measured in the cytoplasm and nucleus and normalized to histone H3 and β-actin. *p < 0.05; **p < 0.01 [vs. NC mimics+LV-NC]; ^†^*P* < 0.05, ^††^*P* < 0.01 [vs. NC mimics+LV-BMP4 or NC mimics+LV-SMAD4]. **e** Cells were treated with vehicle (PBS) or verteporfin and the protein expression of ALP, Col I, and RUNX2 was determined. **f** The activity of ALP was measured. *p < 0.05; **p < 0.01 [vs. PBS]. **g** Mineralization degree determined by staining with Alizarin Red and ALP. Scale bars = 100 μm. Data presented as means ± SEMs. All in vitro experiments: 3 biological replicates × 3 technical replicates

Nuclear translocation of the transcriptional coactivator TAZ is a critical step in transducing SMAD4 signaling [18]; therefore, we next investigated the role of TAZ in relation to miR-1323, BMP4, and SMAD4. Human mesenchymal stromal cells were placed in osteogenic differentiation medium and infected with LV-SMAD4 or LV-BMP4 and co-transfected with miR-1323 mimics. After 7 days of culture, cytoplasmic and nuclear TAZ protein levels were analyzed by Western blot. miR-1323 mimics downregulated nuclear TAZ levels and upregulated cytoplasmic TAZ levels (*p* < 0.01; Fig. 4a–d). BMP4 or SMAD4 overexpression produced an opposite effect on nuclear and cytoplasmic TAZ levels (*p* < 0.01; Fig. 4a–d). Notably, the effects of miR-1323 mimics were rescued by BMP4 or SMAD4 overexpression (*p* < 0.01; Fig. 4a–d).

To further investigate TAZ’s role, human mesenchymal stromal cells were placed in osteogenic differentiation medium and treated with the TAZ inhibitor verteporfin [22, 23] or PBS vehicle. After 7 days of culture, the protein expression of ALP, Col I, and RUNX2 in addition to ALP activity and mineralization were analyzed. Protein levels of ALP, Col I, and RUNX2 were downregulated after TAZ inhibition (*p* < 0.01; Fig. 4e). Furthermore, TAZ inhibition reduced ALP activity (*p* < 0.01; Fig. 4f) and the degree of mineralization (Fig. 4g). Thus, miR-1323 modulates pro-osteogenic TAZ nuclear translocation in mesenchymal stromal cells via regulating BMP4 and SMAD4 expression.

### Osteogenesis upregulated by miR-1323 antagomiR therapy in vivo

**Fig. 5:**
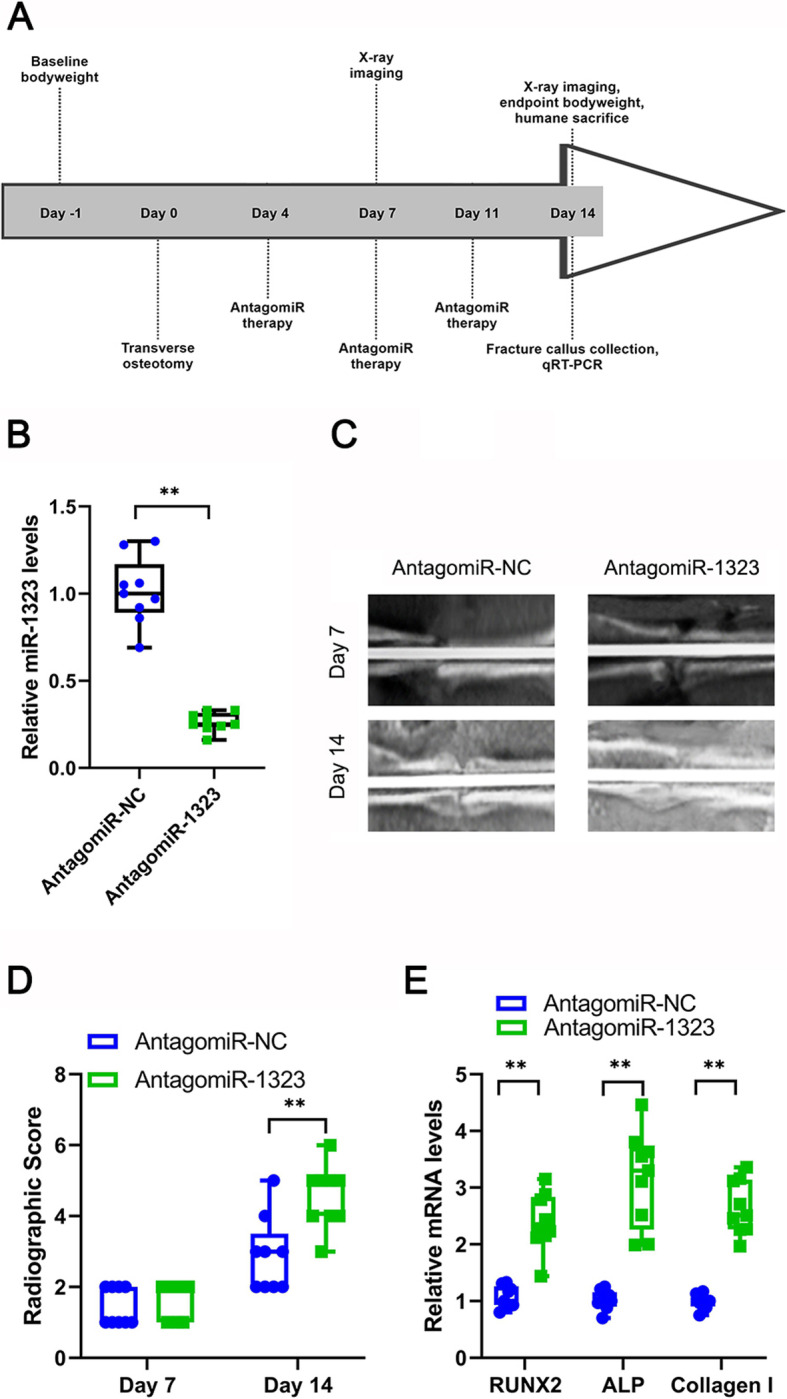
AntagomiR-1323 promotes the formation of bone in the rat femoral fracture model. **a** Schematic overview of the in vivo rat femoral fracture model. AntagomiR-1323 or antagomiR-NC was administered locally in the area of the femur fracture site on days 4, 7, or 11. *n* = 9 rats per group. **b** miR-1323 levels in fracture calluses measured by qRT-PCR on day 14. **c** X-ray imaging of bone formation at the femur fracture sites on days 7 and 14. **d** Radiographic score assessment on days 7 and 14. **e** RUNX2, ALP, and Col I mRNA levels in fracture calluses measured by qRT-PCR on day 14. *p < 0.05, **P < 0.01 [antagomiR-NC vs. antagomiR-1323]. **b**, **d**, **e** Data presented as medians ± interquartile ranges (boxes) and absolute ranges (whiskers)

A rat femur fracture model was established in 18 male SD rats. A negative control (NC) antagomiR (*n* = 9 rats) or miR-1323 antagomiR (*n* = 9 rats) was injected around the fracture to analyze miR-1323’s function in vivo (Fig. 5a). miR-1323 levels in fracture calluses were measured on day 14 by qRT-PCR, confirming successful knockdown by the miR-1323 antagomiR (*p* < 0.01; Fig. 5b). Fracture gaps with hard calluses were readily observed by X-ray on day 7 (Fig. 5c). Notably, on day 14, miR-1323 antagomiR treatment was associated with smaller fracture gaps and larger calluses (Fig. 5c) as well as higher radiographic scores (*p* < 0.01; Fig. 5d). qRT-PCR analysis on day 14 fracture calluses revealed that ALP, Col I, and RUNX2 mRNA levels were increased by miR-1323 antagomiR therapy (*p* < 0.01; Fig. 5e). These findings support the anti-osteogenic function of miR-1323 in vivo.

## Discussion

Atrophic non-union is characterized by the absence of radiological evidence of callus formation within 3 months of fracture [1]. In this scenario, the normal cell responses needed for normal bone remodeling fail to occur [2]. Notably, human mesenchymal stromal cells show reduced cell counts and proliferation in atrophic non-union fractures [3]. However, these cells retain their potential for proliferation, osteoblastic differentiation, and mineralization in atrophic non-union fractures [19]. Utilizing this unused potential can facilitate atrophic non-union fracture healing [3]. We know that human mesenchymal stromal cell osteogenic differentiation may be induced by BMP4, SMAD4, and SMAD4’s downstream mediators TAZ and RUNX2 [17, 18]. In this study, we demonstrated that BMP4 and SMAD4 levels, in addition to the downstream osteogenic markers ALP, Col I, and RUNX2, were downregulated in atrophic non-union specimens. This evidence indicates that osteogenic BMP4/SMAD4 signaling is inhibited in atrophic non-union fractures and is consistent with previous studies showing BMP and SMAD dysregulation in non-union fractures [30, 31].

miRNAs regulate gene expression on a post-transcriptional level and modulate bone formation and remodeling [32]. Recently published microarray data comparing atrophic non-union fractures to normally healing fractures has identified several upregulated miRNAs in atrophic non-union fractures, including miR-27b-3p, miR-381-3p, miR-520d-5p, miR-1323, and miR-4694-3p [16]. We selected miR-1323 for further study based on a focused TargetScan analysis. We found miR-1323 upregulation coupled with BMP4 and SMAD4 downregulation in atrophic non-union specimens, implying miR-1323’s role in regulating atrophic non-union fracture healing. After showing that miR-1323 negatively regulates BMP4 and SMAD4 expression, we demonstrated that overexpressing miR-1323 downregulated osteogenic differentiation of human mesenchymal stromal cells via targeting BMP4 and SMAD4. These in vitro findings were validated in vivo using miR-1323 antagomiR therapy in a rat model of femoral fracture. Our findings mirror those of previous studies that also show miRNA-based modulation of osteo-specific gene expression, ALP activity, and mineralization in mesenchymal stem cells via miRNA-mediated repression of key signaling proteins, such as miR-21’s repression of SOX2 and miR-22’s repression of HDAC6 [33, 34].

Among the members of the SMAD family of proteins, SMAD4 acts as a common (Co-)SMAD that is essential for nuclear transduction of SMAD signaling through SMAD4 complexing with receptor-activated (R-)SMADs (e.g., SMAD2/3, SMAD1/5/8) [35]. Park et al. discovered that SMAD4 binding to the transcriptional coactivator TAZ facilitates TAZ’s nuclear translocation and osteogenic differentiation of mesenchymal stem cells through enhancing nuclear TAZ-RUNX2 complex formation [18]. Consistent with Park et al.’s model, we found that inhibition of miR-1323, which raises SMAD4 levels, facilitates mesenchymal stromal cell osteogenic differentiation via promoting TAZ nuclear translocation. Moreover, this promotion of osteogenic differentiation by miR-1323 inhibition was partially reversed by SMAD4 or BMP4 knockdown.

From a clinical viewpoint, this combined evidence implies that bolstering BMP4 or SMAD4 activity in human atrophic non-union fractures may support mesenchymal precursor cell differentiation and facilitate fracture healing. Indeed, serum SMAD4 levels in senile osteoporotic fracture patients correlate with lower fracture healing durations and lower visual analogue scale (VAS) pain scores [36]. With respect to clinical interventions, preliminary clinical trial evidence suggests that recombinant BMP2 and BMP7, either alone or in combination with autografts, can facilitate non-union healing [37]. Along this vein, translational researchers can also pursue recombinant and antagomiR approaches [38], in combination with conventional autografts or novel osteoinductive autologous bone graft substitutes (ABGS) [37], to promote osteogenic BMP4/SMAD4 activity in vivo.

There are some notable strengths and limitations to this study. Our in vitro experiments employed a human mesenchymal stromal cell model, which directly supports future translational efforts in human patients. As a limiting factor, we employed a normal femoral fracture animal model instead of using an atrophic non-union fracture animal model [39, 40]. Future studies should examine the effects of recombinant BMP4 or SMAD4 therapy as well as miR-1323 antagomiR therapy in animal models of atrophic non-union fracture model to validate our findings.

In conclusion, our study identifies miR-1323’s negative regulation of BMP4 and SMAD4 expression in human atrophic non-union fractures. In vitro, miR-1323 downregulates osteogenic differentiation of human mesenchymal stromal cells via directly targeting BMP4 and SMAD4. Moreover, miR-1323 antagomiR therapy is associated with improvements in femoral fracture healing in vivo. This evidence supports the miR-1323/BMP4 and miR-1323/SMAD4 axes as novel therapeutic targets for atrophic non-union fractures.

## Supplementary information

**Additional file 1: Table S1.** Sequences of oligonucleotides used in this study.

**Additional file 2: Table S2.** Primers used in qRT-PCR experiments.

**Additional file 3: Table S3.** Clinicodemographic characteristics of tibial fracture enrollees (*n* = 10).

**Additional file 4: Figure S1.** Wild-type and mutant SMAD4 3′UTR constructs. (Top) Wild-type miR-1323 binding site 1 in the SMAD4 3′UTR luciferase reporter construct (WT-SMAD4 3′UTR) and the engineered mutant miR-1323 binding site 1 in the SMAD4 3′UTR luciferase reporter construct (MUT-BMP4 3′UTR). (Bottom) Wild-type miR-1323 binding site 2 in the SMAD4 3′UTR luciferase reporter construct (WT-SMAD4 3′UTR) and the engineered mutant miR-1323 binding site 2 in the SMAD4 3′UTR luciferase reporter construct (MUT-BMP4 3′UTR).

**Additional file 5: Figure S2.** Osteogenic differentiation is modulated by miR-1323 via the BMP4/SMAD4 pathway. (A, B) Human mesenchymal stromal cells were infected with lentivirally (LV)-delivered LV-SMAD4, LV-BMP4, or negative control (LV-NC) and co-transfected with either miR-1323 mimics or NC mimics seven days following osteoblastic differentiation induction. SMAD4, BMP4, RUNX2, ALP, and Col I levels were measured with Western blot. (C) ALP activity levels measure with ALP staining. *P < 0.05; **P < 0.01 [vs. NC mimics+LV-NC]; ^†^*P* < 0.05, ^††^*P* < 0.01 [vs. miR-1323 mimics+LV-NC]. Data presented as means ± SEMs. All in vitro experiments: 3 biological replicates × 3 technical replicates.

## Data Availability

The dataset(s) supporting the conclusions of this article are included within the article and its additional files.

## Abbreviations

BMP4: Bone morphogenic protein 4
FBS: Fetal bovine serum
qRT-PCR: Quantitative real-time PCR
H&E: Hematoxylin & eosin
miRNAs: MicroRNAs
SD: Sprague-Dawley
NC: Negative control
