# Supplementary figures and images for: miR-1323 suppresses bone mesenchymal stromal cell osteogenesis and fracture healing via inhibiting BMP4/SMAD4 signaling
Source: J Orthop Surg Res. 2020 Jun 29;15:237. doi: 10.1186/s13018-020-01685-8 (PMC7322887; doi:10.1186/s13018-020-01685-8)

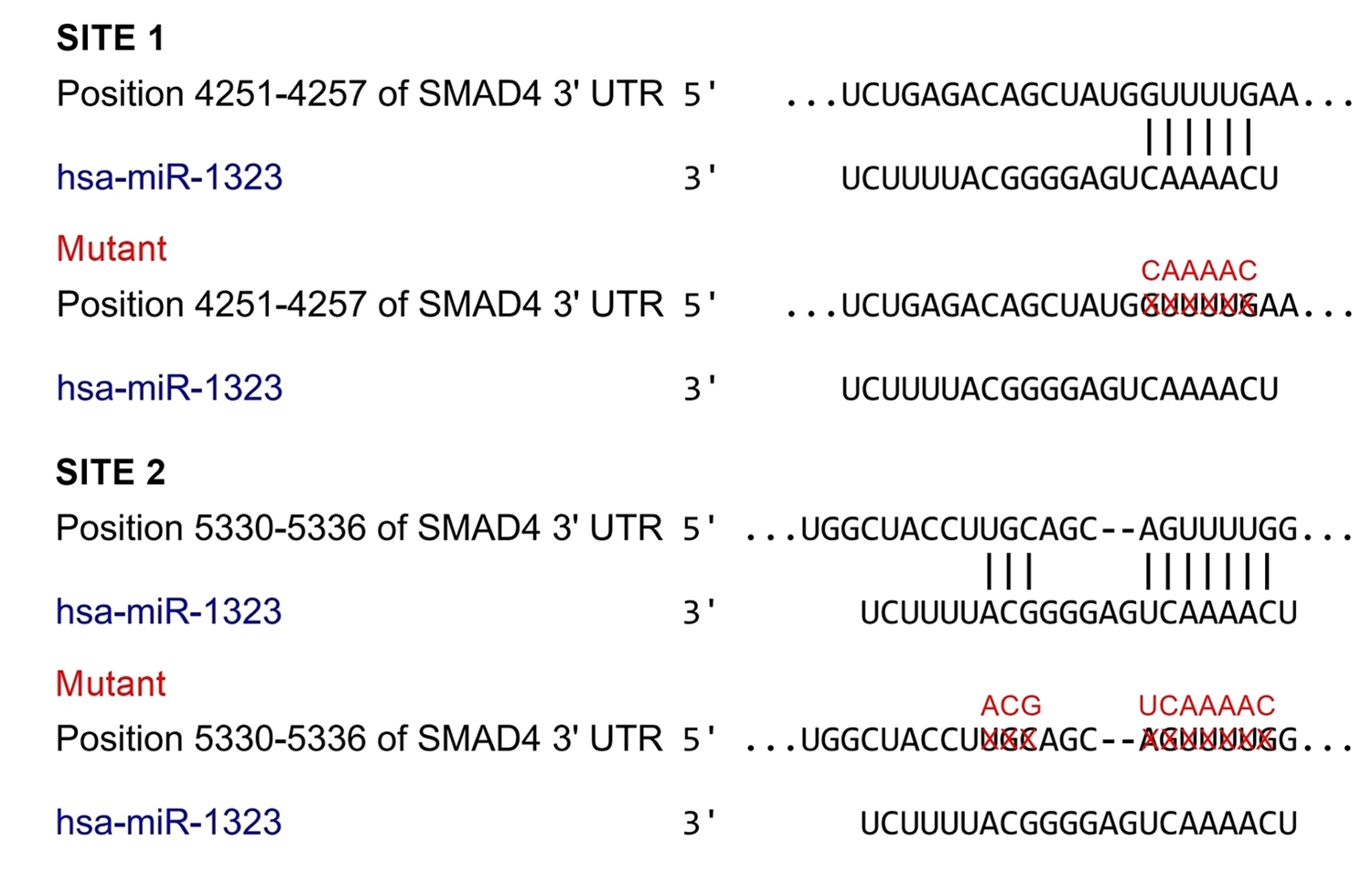

Supplement: Supplementary file 4 — Additional file 4: Figure S1. Wild-type and mutant SMAD4 3′UTR constructs. (Top) Wild-type miR-1323 binding site 1 in the SMAD4 3′UTR luciferase reporter construct (WT-SMAD4 3′UTR) and the engineered mutant miR-1323 binding site 1 in the SMAD4 3′UTR luciferase reporter construct (MUT-BMP4 3′UTR). (Bottom) Wild-type miR-1323 binding site 2 in the SMAD4 3′UTR luciferase reporter construct (WT-SMAD4 3′UTR) and the engineered mutant miR-1323 binding site 2 in the SMAD4 3′UTR luciferase reporter construct (MUT-BMP4 3′UTR). [file 13018_2020_1685_MOESM4_ESM.jpg]

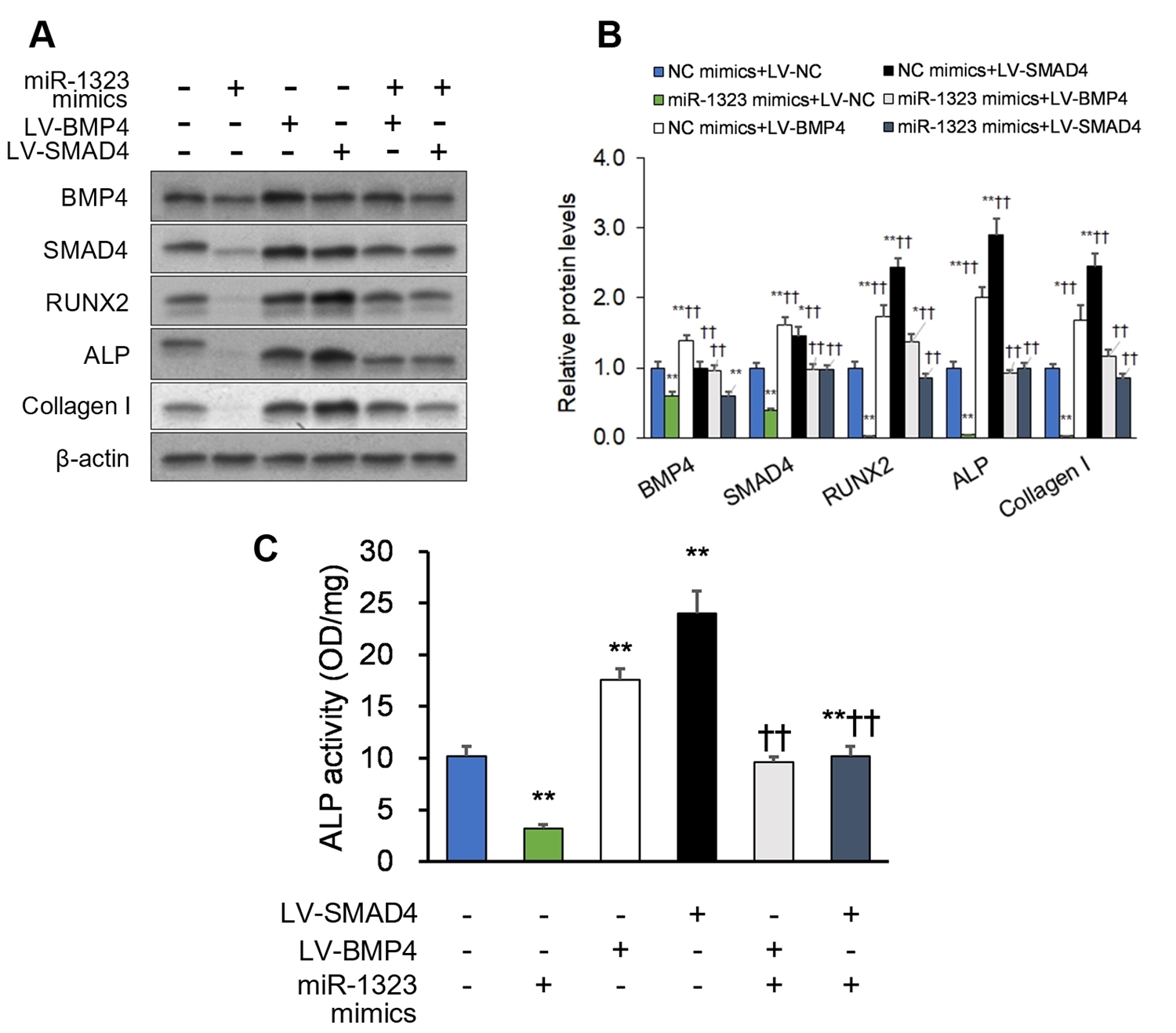

Supplement: Supplementary file 5 — Additional file 5: Figure S2. Osteogenic differentiation is modulated by miR-1323 via the BMP4/SMAD4 pathway. (A, B) Human mesenchymal stromal cells were infected with lentivirally (LV)-delivered LV-SMAD4, LV-BMP4, or negative control (LV-NC) and co-transfected with either miR-1323 mimics or NC mimics seven days following osteoblastic differentiation induction. SMAD4, BMP4, RUNX2, ALP, and Col I levels were measured with Western blot. (C) ALP activity levels measure with ALP staining. *P < 0.05; **P < 0.01 [vs. NC mimics+LV-NC]; †P < 0.05, ††P < 0.01 [vs. miR-1323 mimics+LV-NC]. Data presented as means ± SEMs. All in vitro experiments: 3 biological replicates × 3 technical replicates. [file 13018_2020_1685_MOESM5_ESM.jpg]
